# Poetic representations of post-traumatic stress disorder in cinema - example of ivan’s childhood by andriei tarkovsky

**DOI:** 10.1192/j.eurpsy.2021.1194

**Published:** 2021-08-13

**Authors:** N. Szejko

**Affiliations:** Neurology And Bioethics, Medical University of Warsaw, Warsaw, Poland

**Keywords:** post-traumatic stress disorder, cinema, Tarkovsky, Jungian theory

## Abstract

**Introduction:**

Cinema constitutes an artistic presentation of the spectrum of human emotions and offers a number of examples of artistic vision of posttraumatic stress disorder (PTSD). Cinema of the Russian director, Andriei Tarkovsky, alludes to the complexity of human psycho through poetic narration and cinematography. Particularly, Tarkovsky makes reference to such topics as trauma, depression, melancholy, and madness.

**Objectives:**

The aim of this study is to analyze Tarkovsky’s film “Ivan’s Childhood” from the perspective of psychiatry and psychology with the special attention to the topic of PTSD.

**Methods:**

We identified elements of trauma and PTSD in line with the Diagnostic and Statistical Manual of Mental Disorders (DSM-5). Furthermore, we analyzed manifestations of trauma in “Ivan’s Childhood” according to the trauma complex in Jungian perspective.

**Results:**

The main protagonist of the movie, Ivan, is treated as an archetype of a person exposed to trauma. The traumatic circumstance is the war in which he lost his loved ones. “Ivan’s Childhood” is a poetic presentation of this boy’s struggling to overcome his fears and his personal fight for dignity. Tarkovsky accomplishes it through a series of poetic images in which the director demonstrates flashbacks from Ivan’s life. Based on comparisons to Jung’s model of generic complexes, it is possible to define Ivan’s trauma complex as a set of psychological processes that are archaic and typical, i.e., “archetypal.”

**Conclusions:**

“Ivan’s Childhood” is a moving portrait of a destroyed childhood and subsequent trauma as well as coping mechanisms such as rebellion, alienation, and transference.

